# Testing family-of-origin sensitization: Parent-adolescent conflict, emotional reactivity, and adolescent internalizing psychopathology

**DOI:** 10.1017/S0954579424001779

**Published:** 2024-12-04

**Authors:** Shou-Chun Chiang, Sunhye Bai

**Affiliations:** 1 Department of Human Development and Family Sciences, Texas Tech University, Lubbock, TX, USA; 2 Department of Human Development and Family Studies, The Pennsylvania State University, University Park, PA, USA; 3 Ballmer Institute for Children’s Behavioral Health, University of Oregon, Eugene, OR, USA

**Keywords:** emotional reactivity, family conflict, internalizing problems, sensitization

## Abstract

Building on the sensitization hypothesis, the present work aimed to examine how parent-adolescent conflict might be associated with heightened emotional reactivity to peer conflicts, which in turn shape the development of adolescent internalizing psychopathology. Participants were 108 Taiwanese adolescents between the ages of 18 and 19 (*M*_age_ = 18.53, *SD*_age_ = 0.39; 64% female) who completed baseline assessments, 14-day daily surveys, and 6-month follow-up assessments. Emotional reactivity was measured by calculating the daily association between peer conflict and positive and negative emotions. Results indicated that greater baseline parent-adolescent conflict was associated with higher negative emotional reactivity to peer conflicts, which then predicted increased depressive symptoms and anxiety symptoms 6 months later. Moreover, greater positive emotional reactivity to peer conflicts (i.e., more declines in positive emotions in response to peer conflicts) predicted increased depressive symptoms. Thus, the findings of the current study support and extend the sensitization hypothesis and suggest that parent-adolescent conflict may contribute to family-of-origin sensitization in non-familial, interpersonal contexts. The results have key implications for understanding adolescent developmental psychopathology associated with family conflicts and emotional reactivity.

## Introduction

Adolescence marks an important period of increased risk for the development of psychiatric disorders, due to increased emotional sensitivity to interpersonal stressors (Casey et al., 2019; Dahl & Gunnar, 2009; Green et al., 2021; Somerville et al., 2010). Past research has indicated that emotional reactivity is associated with adolescent psychopathology, including depressive symptoms, anxiety symptoms, and behavioral problems (Chiang et al., 2024a; Herres et al., 2016; Kalvin et al., 2016; Shapero et al., 2016). Family conflict generally predicts greater emotional reactivity. In adolescence, individuals also develop increased emotional sensitivity to specific types of stressors – namely peer stressors. Past research has shown that family conflict and hostile parenting are linked to increased youth sensitivity to peer rejection and problems (Godleski et al., 2019; Guyer et al., 2015). However, limited research examined the direct association between family conflict and emotional reactivity to peer conflict. Moreover, it is unclear whether adolescents from high conflict families would exhibit greater emotional reactivity to peer conflict, which may subsequently predict the future development of internalizing psychopathology. To address these gaps in literature, this study investigated emotional reactivity to peer conflict as a mediator in the association between parent-adolescent conflict and adolescent internalizing problems.

### Sensitization and adolescent psychopathology

The sensitization hypothesis posits that family conflict increases children’s reactivity to future conflicts in the family (Davies & Cummings, 1994). In support of the sensitization hypothesis, past research showed that, instead of habituating to conflicts, adolescents with a history of family conflicts tend to demonstrate heightened emotional reactivity to the later occurrence of conflicts (David & Murphy, 2004; Davies et al., 2021; Goeke-Morey et al., 2013; Pearson et al., 2023). Emotion reactivity, defined as the extent to which an individual reacts emotionally to a stimulus (e.g., family conflict), is one dimension of emotion regulation. In past research, high levels of emotional reactivity have often been characterized by intense reactions to stimuli – reactions so intense that they are emotionally disproportionate or maladaptive (Agako et al., 2022; D’Agostino et al., 2017; Gross & Feldman Barrett, 2011). Emotional security theory (EST) suggests that emotional reactivity reflects adolescents’ concerns about their security in the family (Davies et al., 2006) and such emotional reactivity is linked to the development of internalizing and externalizing problems (Buehler & Welsh, 2009; Davies et al., 2012). Specifically, emotional reactivity serves as a mediator that bridges the association between family conflict and maladjustment (Davies et al., 2021). In an attempt to preserve a sense of emotional security, children with high emotional reactivity are able to instantly identify potential threats of conflicts, and access psychophysiological resources to effectively adapt to them (Davies et al., 2016). However, such heightened status of arousal and vigilance would lead to prolonged and dysregulated distress (Davies et al., 2006). Thus, EST states that family conflict may undermine adolescents’ emotion regulation and that heightened emotional reactivity is one manifestation.

Much of the research on EST has focused on interparental conflict. Past research has shown that sensitization would also emerge from general family conflict and parent-adolescent interactions (Chiang et al., 2023, 2024a; Fosco & Grych, 2007; Herres et al., 2018). A history of family conflicts, either in the family in general or between parents and adolescents, would contribute to youth’s heightened emotional reactivity. For instance, youth from families with high levels of conflicts (e.g., we fight a lot in our family) reported lower emotional insecurity in the family (Cummings et al., 2016). In daily life, adolescents were angrier on days when they reported conflict with their parents (LoBraico et al., 2020). These findings suggest that adolescents may experience sensitization to parent-adolescent conflict, in addition to interparental conflict. Thus, adolescents who historically have a lot of conflict with their parents may be more reactive to future conflicts with their parents, which may then signal greater vulnerability to psychopathology. Building on these findings, the present study aims to extend the understanding of sensitization to conflict by investigating whether global parent-adolescent conflict is linked to adolescent emotional reactivity to conflicts in a different close relationship – peers – which in turn may be associated with adolescent psychopathology.

### Family-of-origin sensitization to peer conflict

Family-of-origin sensitization refers to the concept that individuals exposed to destructive family conflict are more likely to view conflict in close relationships outside of family as a threat to their own emotional security, stimulating above-average emotional reactivity to conflict in peer or romantic relationships (Cook et al., 2013). That is, family conflicts may sensitize youth to conflicts in non-familial contexts, such as peer conflicts; heightened emotional reactivity to peer conflict can mediate the link between parent-adolescent conflict and subsequent psychological problems, especially in young adulthood. As a result, family serves as the original source of sensitization, exacerbating emotional reactivity in other relational contexts. Peer relationships are at the center of adolescents’ daily lives and well-being (Brown & Bakken, 2011; Chiang et al., 2024b; La Greca & Harrison, 2005; Poulin & Chan, 2010). They are particularly important during the transition to college, when adolescents spend less time with family members and tend to live with peers and friends. Research has documented that family discords are associated with the quality of peer relations and friendships (Allen et al., 2002; Chung et al., 2011; Schudlich et al., 2004) but little research has documented the presence of family-of-origin sensitization and examined whether emotional reactivity to peer conflict is a mediating mechanism linking parent-adolescent conflict and adolescent psychological problems. Past research shows that exposure to high levels of conflict in one’s family of origin is detrimental to future relationships with peers. Adolescents exposed to interparental conflict perceived such conflicts as more threatening to their own well-being and the family, which increased the risk for social anxiety symptoms and subsequent lower levels of peer support (Weymouth et al., 2019). Similarly, in a 10-year longitudinal study, interparental conflict in childhood predicted increased emotional insecurity 5 years later in adolescence, and such insecurity predicted decreases in adolescent friendship affiliation (e.g., warmth, intimate disclosure) (Davies et al., 2018). The current study specifically tests family-of-origin sensitivity by testing the association between parent-adolescent conflict and emotional reactivity to peer conflicts in older adolescents.

Emotional reactivity to peer conflict may serve as a mediating mechanism between parent-adolescent conflict and adolescent psychopathology. The quality of early parent-child relationships shapes youth’s internal working models, which reflect their representations of both themselves and others. Insecure attachment styles lead to negative internal working models, which impede the development of future healthy relationships – an essential protective factor that promotes mental wellness (Herres et al., 2018; Kerns & Brumariu, 2014; Michiels et al., 2008; Verschueren et al., 1996; Wang et al., 2021). EST extends attachment theory and posits emotional reactivity as a developmental mechanism that links family conflict and psychopathology. Family conflicts lead to individuals becoming more sensitive and reactive to conflicts with peers, and viewing the conflict as threatening to their own and the relationship’s health (Cook et al., 2018). In turn, the frequent activation of strong negative emotions and the declining relationship quality may increase risk for internalizing problems. Cook et al. (2013) found emotional reactivity in the context of interparental conflict was associated with reactivity to conflict in later friendship and romantic relationships. The findings suggest that family conflict may result in greater emotional reactivity to non-familial interpersonal stressors. Together, we argue that past family conflicts may put adolescents at greater risk for sensitization to peer conflict. This increased emotional reactivity, in turn, may contribute to poor mental health (Kleiman et al., 2014; Spear, 2009).

Family-of-origin sensitization is developmentally appropriate to examine during the transition to young adulthood, when many youths are physically leaving their family of origin to live among peers. Relatedly, this transition is a key developmental milestone, and a time when youth gain significant levels of independence and form new relationships, often with same-aged peers. Whereas parents are primary partners in interpersonal emotion regulation during childhood and adolescence, peers take on this role during the transition to college, due to their greater physical. All the while, biological maturation underlying the development of emotion regulation capacity and autonomy from peer influences is still underway, well into mid-20s (Andrews et al., 2021; Sawyer et al., 2018).

### Assessing emotional reactivity in daily life

One remaining gap is characterizing emotional reactivity in naturalistic settings using intensive longitudinal methods. Most research utilizes one-time questionnaires or laboratory observations to examine emotional reactivity to family or interpersonal discords. For instance, researchers used the well-validated scales, such as Emotional Reactivity Subscale of the Security in the Interparental Subsystem scale (SIS, Davies et al., 2002), to evaluate adolescents’ emotional reactivity to interparental conflict (Buehler & Welsh, 2009; Cook et al., 2013; Schermerhorn et al., 2007). Other studies measured emotional reactivity elicited from structured interviews and tasks (Davies et al., 2012; Pearson et al., 2023). Although these assessments provide robust evidence of one’s general level of emotional reactivity, intensive longitudinal methods such as daily diaries and ecological momentary assessment can further complement the existing literature by capturing emotional responses to specific types of stressful events as they naturally occur with reduced recall bias and high ecological validity (Bolger et al., 2003; Laurenceau & Bolger, 2005). Thus, the current investigation operationalizes emotional reactivity as the same-day association between peer conflicts and adolescent positive and negative emotions.

Additionally, limited research considers both positive and negative emotional reactivity. Most studies have focused on negative emotions (e.g., sad, fearful, angry) (Cook et al., 2018; Davies et al., 2012; Kelley et al., 2016). Nevertheless, positive emotional reactivity may also provide unique information on adolescents’ emotional responses that are largely unknown in literature. Positive emotions have unique contributions to the development of adaptive capacity and personal resources, including cognitive (e.g., enhancing learning performance), physical (e.g., health conditions, psychological (e.g., resilience), and social (maintaining relationships) (Gilbert, 2012; Taylor et al., 2023). Positive emotional reactivity may represent a strong indicator of emotion dysregulation with significant declines in positive emotions in the face of distress and negative events.

### The present study

**Figure 1.**
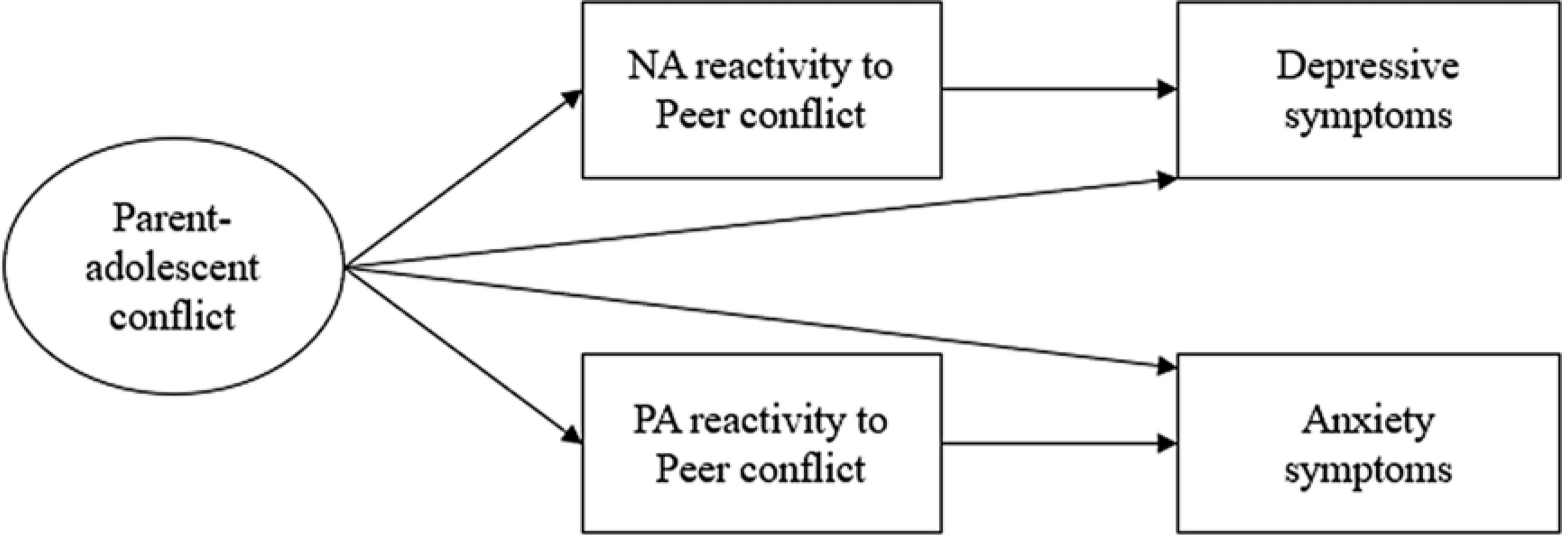
Conceptual model of family-of-origin sensitization and mental health problems.

The present study examined the association between parent-adolescent conflict, emotional reactivity to peer conflict, and adolescent mental health problems, and tested the mediating role of emotional reactivity to peer conflict in the link between parent-adolescent conflict and mental health problems. This study extended prior research by using a daily diary approach with a 6-month follow-up assessment. Rather than measuring emotional reactivity using a single administration questionnaire, this study captured daily emotional reactivity by examining within-person relations between peer conflict and emotions. In addition, our Taiwanese sample differs from the past predominantly North American and European samples. Given the cultural emphasis on family relations in collectivistic societies, testing family-of-origin sensitization in this sample may enrich our understanding of the EST and sensitization hypothesis. As shown in Figure 1, we had three hypotheses regarding the family-of-origin sensitization. First, we aimed to evaluate the associations between parent-adolescent conflict and emotional reactivity to peer conflict, including positive and negative emotional reactivity. We hypothesized that higher parent-adolescent conflict would be associated with greater positive and negative emotional reactivity to peer conflict, testing the hypothesis of family-of-origin sensitization. Second, we examined whether emotional reactivity to peer conflict was prospectively associated with mental health problems as indicated by depressive symptoms and anxiety symptoms. We hypothesized that greater emotional reactivity would be associated with more depressive symptoms and anxiety symptoms. Next, we evaluated whether emotional reactivity to peer conflict would mediate the prospective relationships between parent-adolescent conflict and mental health problems.

## Method

### Participants

Participants consisted of 108 Taiwanese first-year college students between ages 18 and 19 (*M*_*age*_ = 18.53, *SD*_*age*_ = 0.39; 64% female) recruited in Taiwan. All participants were born in Taiwan and the majority (96%) of their parents were Taiwanese (3% Chinese, 1% Southeast Asian). To be eligible for participation, participants needed to meet the following inclusion criteria: (a) access to mobile devices most of time to complete online surveys; (b) living with parents most of time before entering college; (c) contact with parents at least once a month (35% currently living with parents). Participants reported parent education level ranging from below high school degree (6%), high school degree (23%), college degree (59%), and graduate degree (12%), as well as family income ranging from low-income (4%), middle-to-low (17%), middle (63%), upper-to-middle (14%), and rich (2%). Baseline and daily diary surveys were conducted between December 2022 and January 2023, and the follow-up assessments were conducted in June 2023.

All study procedures were approved by the affiliated University’s Institutional Review Board. Participants were recruited by referral and snowball sampling from three universities in north and central Taiwan. The research team reached out to college faculty who invited eligible students to enroll in the study via classroom announcements. The research team explained research goals and procedures to interested participants. After informed consent, participants completed a general questionnaire about demographic information, family and peer relationship, health behaviors, and well-being. Starting from Monday, participants completed 14-day ecological momentary assessment four times per day. Given the focus on peer conflict and emotion, this study utilized evening surveys, which asked about peer conflict and positive and negative emotions. Each night, participants received daily surveys through emails or apps with person-specific links at 7 pm. The compliance with daily surveys were excellent (99.5% of total prompts completed). Participants were compensated with $35 USD for their participation. Six months later, the participants completed the follow-up assessments and completed questions about their mental health. All participants completed baseline and follow-up assessments.

### Measures

#### Parent-adolescent conflict

Adolescents rated the extent to which they had conflicts with parents on three items adapted from the Parent-Adolescent Relationship Scale in the Taiwan Youth Project, a large-scale, longitudinal panel study in Taiwan (Yi et al., 2009): “I argued with my parent a lot” “My parent and I were angry toward each other,” and “I had conflicts with my parent.” Past studies have shown that the parent-adolescent conflicts subscale has good validity and reliability (Cheng & Wang, 2021; Chiang & Bai, 2022; Jou et al., 2010). Items were rated on a five-point scale ranging from 1 (*strongly disagree*) to 5 (*strongly agree*). Internal reliability (Cronbach’s *α*) was 0.81.

#### Depressive symptoms

The Patient Health Questionnaire-9 (PHQ-9; Kroenke et al., 2001) was used to assess depressive symptoms at the baseline and follow-up surveys. PHQ-9 has excellent psychometric validity and reliability for assessing depressive symptoms among clinical and community samples (Fonseca-Pedrero et al., 2023; Gao & Liu, 2024; Richardson et al., 2010). Adolescents were asked to report how often they have experienced depressive symptoms during the past two weeks with nine items such as “little interest or pleasure in doing things,” “feeling down, depressed, or hopeless,” and “trouble falling or staying asleep, or sleeping too much.” Items were rated on a four-point scale ranging from 0 (*not at all*) to 3 (*nearly every day*) and averaged. Internal reliability (Cronbach’s *α*) was 0.86 at baseline and 0.85 at follow-up. Higher scores indicated greater depressive symptoms.

#### Anxiety symptoms

The Generalized Anxiety Disorder-7 (GAD-7; Spitzer et al., 2006) assessed the severity of anxiety symptoms. Research has shown that GAD-7 has great psychometric properties (e.g., specificity, sensitivity) for detecting the severity of youth anxiety symptoms (Mossman et al., 2017; Tiirikainen et al., 2019). Adolescents were asked to report how often they have experienced anxiety symptoms during the past two weeks on seven items such as “feeling nervous, anxious, or on edge,” “worrying too much about different things,” and “trouble relaxing.” Items were rated on a four-point scale ranging from 0 (*not at all*) to 3 (*nearly every day*) and averaged. Internal reliability (Cronbach’s *α*) was 0.84 at baseline and 0.87 at follow-up. Higher scores indicated greater anxiety symptoms.

#### Daily peer conflict

Adolescents completed two items assessing peer conflict modified from past daily diary studies (Chiang et al., 2024b): “I argued with my friend(s) today” and “I did not get along with my friend(s) today.” Adolescents were asked to rate how much they experienced peer conflict during the day on a five-point scale from 1 (*not at all*) to 5 (*a lot*). Given that peer conflict was assessed daily, frequency can be inferred from a summative score (e.g., number of days when the individual selected >2 on the Likert scale). Nonetheless, the focus of the current study was on same-day responses to peer conflict, and the Likert scale yielded greater variability on the day-level predictor. Reliability was good for within-person (*R*_C_ = .80) and between-person (*R*_1F_ = .89) levels. Items were averaged to create a single score of peer conflict.

#### Daily positive and negative emotion

Adolescents reported their daily emotions on ten items adapted from the Positive and Negative Affect Schedule-Short Form (PANAS-SF, Thompson, 2007). We assessed positive emotion with five items (i.e., determined, active, attentive, inspired, alert) and negative emotion with five items (i.e., nervous, sad, hostile, ashamed, afraid). Adolescents were asked to rate their emotion during the day on a five-point scale from 1 (*not at all*) to 5 (*a lot*). Reliability was good for within-person (positive: *R*_C_ = .89; negative: *R*_C_ = .82) and between-person (positive: *R*_1F_ = .94; negative: *R*_1F_ = .90) levels. Items were averaged to create daily scores of positive and negative emotions.

#### Analytical plan

This study examined the association between parent-adolescent conflict, emotional reactivity to peer conflict, and psychopathology indicated by depressive and anxiety symptoms. To test the hypotheses, we conducted two-step analytical procedures using R. In the first step, we captured daily emotional reactivity to peer conflict using two Multilevel Models (MLM): one estimating negative emotions and the second estimating positive emotions. Consistent with past research (Mroczek et al., 2015; Ong et al., 2020; Santee & Starr, 2022), we calculated emotional reactivity in MLM by deriving the within-person slopes of the daily associations between person-mean-centered peer conflict and positive and negative emotions. Specifically, each person has within-person random intercepts and slopes wherein the intercepts represent the average levels of emotions, and the slopes indicate the unique association between peer conflict and emotions on a given day. These slopes reflect the degree to which changes in individual’s peer conflicts predict the corresponding changes in positive and negative emotions across the daily diary period, suggesting each individual’s emotional reactivity to peer conflict. Based on these, we derived Empirical Bayes estimates of each individual’s slope (Chiang & Bai, 2024; Santee & Starr, 2022), which quantified each adolescents’ unique relationship between peer conflict and positive and negative emotions as an index of emotional reactivity to peer conflict. Thus, these estimates documented the levels of emotional reactivity for each person (i.e., between-person index). For positive emotional reactivity only, we recoded the scores (multiplying by −1) so that a higher score indicates that the individual experienced greater declines in positive emotions on days when they experienced more peer problems than was usual for them (Bai & Repetti, 2018). Higher emotional reactivity refers to greater increases in Negative Emotion (NE reactivity) or greater declines in Positive Emotion (PE reactivity).

In the second step, we included the indices of NE and PE reactivity in a single Structural Equation Model (SEM) to test their mediation effect in the relationship between parent-adolescent conflict and psychopathology. SEM was conducted using lavaan package in R (Rosseel, 2012) with full information maximum likelihood method. We included a latent variable of parent-adolescent conflict (indicated by the three items) and NE and PE reactivity scores in a SEM model with depressive symptoms and anxiety symptoms as the dependent variables. Residual errors of positive emotional reactivity and negative emotional reactivity were allowed to correlate. Given that Chi-square (χ^2^) is vulnerable to sample size, we evaluated the model fit using the following fit indexes: Root Mean Square Error of Approximation (RMSEA), Comparative Fit Index (CFI), and the Standardized Root Mean Square Residual (SRMR). The model fit was considered acceptable when RMSEA was below 0.08, CFI was above 0.95, and SRMR was below 0.05 (Bollen, 1989; Hu and Bentler, 1999). To evaluate the mediation effect of emotional reactivity, we utilized a bootstrap procedure to test the indirect effect (MacKinnon et al., 2002). Following the recommendations (Mallinckrodt et al., 2006; Shrout& Bolger, 2002), we used 95% bias-corrected confidence intervals with 5,000 bootstrapping resamples to calculate the mediation effect in the analyses.

## Results

Descriptive statistics and correlations were presented in Table 1. The within-day correlations between peer conflict and positive and negative emotions were −0.10 (*p* < .001) and 0.34 (*p* < .001), respectively. The results of MLM analyses showed that, for the average adolescent, more peer conflicts were associated with lower positive emotions (*b* = −0.37, *p* < .001) and higher negative emotions (*b* = 0.49, *p* < .001) on that day. Further there was significant individual variability in PE and NE reactivity to peer conflicts, indicated by significant likelihood ratio test of variance components (*p* < .001). We then extracted these slopes to represent the unique association between peer conflict and emotions for each adolescent in SEM models. To test our main hypotheses, we specified one structural model estimating pathways between global parent-adolescent conflict, PE and NE reactivity to peer conflict, and adolescent depressive symptoms and anxiety symptoms. We conservatively included baseline levels of depressive symptoms and anxiety symptoms.

**Figure 2.**
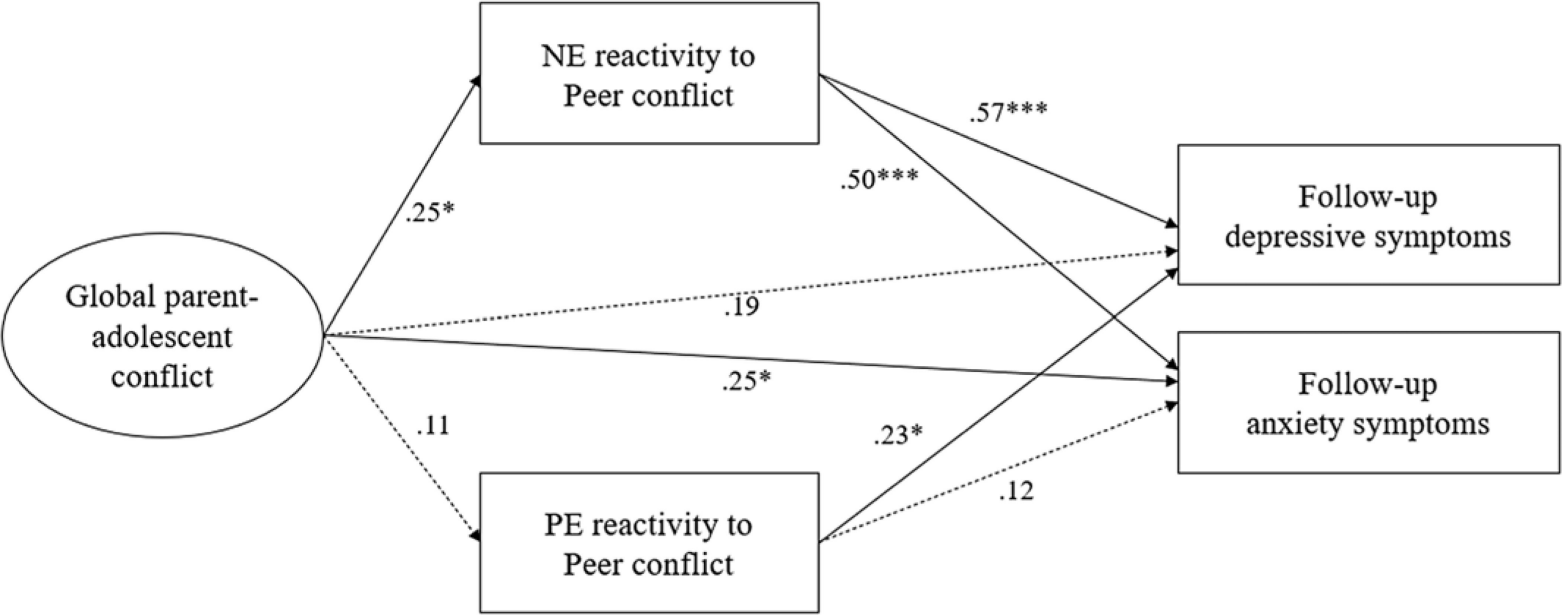
Structural equation modeling of the association between global parent-adolescent conflict, emotional reactivity, and depressive symptoms and anxiety symptoms. Paths were standardized coefficients. The model controlled for outcomes at baseline. *p < .05, **p < .01, ***p < .001.

Given recent work suggesting that a significant association between the predictor and the outcome is not a prerequisite for mediational analyses (Hayes, 2009; O’Rourke & MacKinnon, 2018), we tested the integrated model in which emotional reactivity to peer problems mediated the association between global parent-adolescent conflict and depressive symptoms. The mediational model fit the data well, χ^2^ = 89.45, *p* < .01, RMSEA = 0.04, CFI = 0.96, SRMR = 0.04. As shown in Figure 2, higher level of global parent-adolescent conflict was associated with greater NE reactivity to peer conflict (*b* = .25, *p* < .05) but not associated with PE reactivity to peer conflict (*b* = .11, *p* > .05). Next, greater NE reactivity to peer conflict was associated with more depressive symptoms (*b* = .57, *p* < .001) and anxiety symptoms (*b* = .50, *p* < .001). PE reactivity to peer conflict was also associated with depressive symptoms (*b* = .23, *p* < .05) but not associated with anxiety symptoms (*b* = .12, *p* > .05). To examine the potential mediation effect of NE reactivity, we used bootstrapping method with 5,000 resamples to calculate the 95% confidence intervals around the indirect effect. The indirect effect from global parent-adolescent conflict to depression symptoms via NE reactivity to peer conflict was significant (*b* = .12, *p* < .05; 95% CI [0.04, 0.22]), indicating that NE reactivity mediated the prospective association between global parent-adolescent conflict and depressive symptoms. To test whether NE reactivity fully or partially mediated the association, we conducted a constrained model that evaluated changes in model fit when limiting the path from global parent-adolescent conflict to depressive symptoms as zero. The constrained model did not significantly differ from the original model, Δχ^2^(1) = 1.42, *p* > .05, suggesting that NE reactivity full mediated the association between global parent-adolescent conflict and depressive symptoms.

Next, the results showed that the indirect effect of global parent-adolescent conflict on anxiety symptoms via NE reactivity to peer conflict was significant (*b* = .13, *p* < .05; 95% CI [0.03, 0.19]), indicating that NE reactivity mediated the prospective association between global parent-adolescent conflict and anxiety symptoms. We constrained the path from global parent-adolescent conflict to anxiety symptoms as zero to test whether NE reactivity partially or fully mediated the prospective association. However, the constrained model significantly differed from the original model, Δχ^2^(1) = 4.20, *p* < .05, suggesting that NE reactivity only partially mediated the association between global parent-adolescent conflict and anxiety symptoms.

## Discussion

Guided by the sensitization hypothesis (Cummings & Davies, 2002; Goeke-Morey et al., 2013), this study extends previous findings by examining the mediating effect of emotional reactivity to peer conflict on the prospective associations between global parent-adolescent conflict and adolescent psychopathology. The study uniquely assessed both PE and NE emotional reactivity using a sample of adolescents who reported the daily levels of peer conflict and positive and negative emotions. Findings showed that higher global parent-adolescent conflict was associated with greater NE reactivity to peer conflict, which in turn was associated with more depressive symptoms and anxiety symptoms 6 months later. Mediation analyses showed that NE reactivity to peer conflict mediated the prospective association of global parent-adolescent conflict with depressive symptoms and anxiety symptoms. Moreover, PE reactivity to peer conflict was associated with more depressive symptoms. Our results revealed that sensitization to interpersonal conflicts may originate from parent-adolescent conflict and influence adolescents’ negative emotional reactivity to conflicts outside the family.

The findings extend past studies that primarily focused on sensitization to specific types of conflict – particularly, interparental conflict (e.g., Davies et al., 2012; Goeke-Morey et al., 2013). We found that youth who experienced more conflict with their parents reported greater negative emotions in response to peer conflict, which in turn predicted higher levels of depression and anxiety symptoms. Youth with greater parent conflict may perceive peer conflicts as more threatening or indicative of poor relational quality, leading to heightened negative emotions (Cook et al., 2013). It is also possible that repeated exposure to family conflict – a form of chronic stress – undermines the youth’s ability to react and recover adaptively in the face of future stressors, such as peer conflict. Whether through an increased sense of poor relationship quality or dysregulation of the affective stress response system, parent-child conflict increases the risk of depression and anxiety (Byrd et al., 2022; Kretschmer et al., 2016; Michiels et al., 2008). This pattern aligns with previous research showing that greater NE reactivity predicts the future development of psychopathology (Davies et al., 2012; Santee & Starr, 2022; Stepp et al., 2016). Furthermore, NE reactivity to peer conflict mediated the prospective associations between global parent-adolescent conflict and depressive and anxiety symptoms. These findings support the sensitization hypothesis and emotional security theory, which propose that family conflict exacerbates children’s emotional reactivity, thereby increasing their vulnerability to psychological problems (Cummings & Davies, 2002; Goeke-Morey et al., 2013; Pearson et al., 2023). The results suggest that sensitization originating within the family can heighten adolescents’ NE reactivity in non-familial contexts, supporting the family-of-origin sensitization hypothesis. This study is the first to find that parent-adolescent conflict was associated with heightened emotional reactivity to peer conflict, which then predicted future risk of depressive symptoms and anxiety symptoms among adolescents. Thus, conflict with parents appears to undermine emotional security in new relationships that adolescents form throughout young adulthood, further increasing risk for psychopathology.

Moreover, the findings suggest that greater PE reactivity to peer conflict (i.e., more decreases in positive emotions) was prospectively associated with increases in depressive symptoms, offering new insights into the importance of positive emotions. Positive emotions support adaptive responses to distress and promote coping and regulation in stressful situations (Taylor et al., 2023). It is noteworthy that the evaluation of positive emotional responses adds to the existing literature, which has predominantly focused on negative emotions. The majority of studies have concentrated on NE reactivity, often overlooking the potential impact of PE reactivity on psychological well-being (e.g., Davies et al., 2002; Eisenberg et al., 1994; Nock et al., 2008). A meta-analysis across 58 published papers exploring the relationship between child maltreatment and the development of emotion reactivity revealed a notable absence of investigations into experiences of positive emotions (Lavi et al., 2019). This gap in the literature underscores the limited exploration of PE reactivity, with research in its early stages (Chiang et al., 2024a). Consequently, our findings contribute to the literature on emotional reactivity by finding that PE reactivity to peer conflict predicts the emergence of depressive symptoms, thereby advancing our understanding of the nuanced interplay between positive emotions and psychological outcomes.

Our finding that NE reactivity plays a more prominent role in family-of-origin sensitization is aligned with prior work on emotion dynamics and psychological well-being. Emotion dynamics, defined as patterns, changes and fluctuations in emotional states over multiple points in time, is associated with individual differences in their psychological well-being; this association is much stronger for negative emotions than positive emotions, suggesting that negative emotion dynamics constitute a more significant target of clinical interventions (Houben et al., 2015). Consistent with the emotion security theory, parent-child conflict in the family of origin likely sensitizes individuals to experience more intense and overwhelming negative emotions when their emotion security is threatened (Buehler & Welsh, 2009; Davies et al., 2012). We found that PE reactivity predicted more depressive symptoms, consistent with the tripartite model of anxiety and depression, wherein distress (i.e., negative emotion) characterizes both anxiety and depression and the absence of positive emotion differentiates depression from anxiety (Clark & Watson, 1991).

Study findings have translational implications. Specifically, they point to several distal and proximal targets interventions for the prevention of depression and anxiety during the transition to college. First, as documented in prior research, high levels of conflict between parent and adolescents pose risk for more depression and anxiety symptoms during the transition to young adulthood. Existing programs to foster healthy parent-adolescent relationships (Van Ryzin et al., 2016), are important for youth mental health. One mechanism by which parent-adolescent conflict contributes to depression and anxiety symptoms is via increased negative emotion reactivity to peer conflict in non-familial contexts. Thus, a more proximal prevention interventions for the reduction of depression and anxiety symptoms in young adulthood may include healthy relationship development with an emphasis on peers and romantic partners, and negative emotion regulation.

This study had several methodological strengths. By using the daily diary design, we assessed adolescents’ emotional reactivity through daily reports of peer conflicts and emotions, which reduced recall bias and provided a more objective evaluation of emotional reactivity. Next, the longitudinal design allowed us to examine the prospective association over six months while controlling for baseline levels of the outcomes. Third, the high compliance rate across baseline, daily diary, and follow-up assessments in the study can minimize potential attrition biases because of participant attrition and missing data.

Despite these strengths, several limitations of this study should be noted and addressed in future research. First, the results may not be generalizable to other families with diverse racial/ethnic backgrounds. However, this study represents one of the few studies that investigated sensitization hypothesis in non-Western samples, and we found evidence in support of the sensitization hypothesis in Taiwanese adolescents. Future studies could expand on the sensitization hypothesis by investigating cultural influences on the pathways between family conflict, emotional reactivity, and adolescent psychopathology. Second, our sample was not recruited from a clinical population, potentially constraining the applicability of the findings to youths with greater vulnerability to psychiatric illness. Nonetheless, these findings may have relevance for adolescents at early risk of psychopathology during the transition to college when life stressors increase and mental health deteriorates (Fruehwirth et al., 2023). Third, it is important to acknowledge that the study relied on adolescent reports to test our hypotheses. Future research would benefit from applying multi-informant designs to examine family conflicts and adolescent emotional reactivity from diverse perspectives. Fourth, we only focused on the level of peer conflicts, which may not adequately capture the full scope of peer conflicts such as the frequency, intensity, and resolutions of peer conflicts. Examining various aspects of peer conflicts is an important next step for future research, as investigating the differences in peer conflicts will further clarify whether adolescents would exhibit different levels of emotional reactivity. Fifth, emotional reactivity was defined as maladaptive responses to stressors in the current study, which assumed a linear association between emotional reactivity and mental health problems^1^. However, a mild level of emotional reactivity to significant stressors may be adaptive and normative reactions; thus, future research should further investigate whether emotional reactivity to stressors would exhibit as a curvilinear risk factor for predicting adolescent psychopathology. Finally, we considered the relationship between parent-adolescent conflict and adolescent emotional reactivity and psychopathology; however, recent studies have supported the bidirectional links between family systems in which adolescent characteristics would also influence the quality of parent-adolescent relationships (Chiang & Bai, 2022; Cortes-Garcia et al., 2019). Assessing the nature of bidirectionality in sensitization hypothesis is an important direction for future research.

In conclusion, this study makes theoretical and empirical contributions to the extant literature. Current theoretical frameworks of sensitization hypothesis primarily focus on emotional reactivity to interparental conflict. In this study, we provided novel evidence of how conflicts with parents increase adolescents’ NE reactivity to peer conflict, which then predicted increase in depression and anxiety symptoms. The findings also suggest that depressive symptoms are particularly related to PE reactivity to peer conflict, which is often neglected in studies of emotional reactivity or sensitization hypothesis. Moreover, this is the first known study to examine the unique effects of PE and NE reactivity to peer conflict on depression and anxiety symptoms, thus underscoring the importance of specifying both positive and negative experiences of emotions as predictors of developmental outcomes. Regarding translational contributions, the findings highlight the increased negative emotions in response to peer conflict is a target for preventive interventions that aim to reduce the adverse impacts of parent-adolescent conflict.

## Figures and Tables

**Table 1.** Descriptive statistics and correlations

	1.	2.	3.	4.	5.	6.	7.	8.

1. Parent-adolescent conflict								

2. PE reactivity	.12							

3. NE reactivity	.22*	.42***						

4. Positive emotions	−.22*	−.69***	−.11					

5. Negative emotions	.40***	.27**	.51***	−.26**				

6. Peer conflicts	.27**	.04	.24*	−.09	.55***			

7. Depressive symptoms	.30**	.27*	.42***	−.27**	.46***	.29**		

8. Anxiety symptoms	.35***	.03	.46***	−.20*	.45***	.24*	.70***	

Mean	2.25	−0.08	0.07	3.08	1.81	2.84	0.93	0.87

SD	0.86	0.11	0.12	0.68	0.70	1.06	0.51	0.67

* *p* < .05,

** *p* < .01,

*** *p* < .001.

## Footnotes

1 We explored the potential curvilinear association between emotional reactivity and mental health problems, and the results showed that both NE and PE reactivity were not associated with any outcomes. Thus, the findings support our operationalization of emotional reactivity as a linear risk factor that reflect maladaptive regulatory problems in adolescents.
